# Supplementary material for: The mTORC1-mediated activation of ATF4 promotes protein and glutathione synthesis downstream of growth signals
Source: eLife. 2021 Mar 1;10:e63326. doi: 10.7554/eLife.63326 (PMC7997658; doi:10.7554/eLife.63326)
Supplement: Transparent reporting form [file elife-63326-transrepform.docx]

***eLife’s* transparent reporting form**

We encourage authors to provide detailed information *within their submission* to facilitate the interpretation and replication of experiments. Authors can upload supporting documentation to indicate the use of appropriate reporting guidelines for health-related research (see [EQUATOR Network](http://www.equator-network.org/%20)), life science research (see the [BioSharing Information Resource](https://biosharing.org/)), or the [ARRIVE guidelines](http://www.plosbiology.org/article/info:doi/10.1371/journal.pbio.1000412) for reporting work involving animal research. Where applicable, authors should refer to any relevant reporting standards documents in this form.

If you have any questions, please consult our Journal Policies and/or contact us: [editorial@elifesciences.org](mailto:editorial@elifesciences.org).

**Sample-size estimation**

- You should state whether an appropriate sample size was computed when the study was being designed
- You should state the statistical method of sample size computation and any required assumptions
- If no explicit power analysis was used, you should describe how you decided what sample (replicate) size (number) to use

Please outline where this information can be found within the submission (e.g., sections or figure legends), or explain why this information doesn’t apply to your submission:

Sample size was determined based on previous studies using standard protocols, without a specific method for sample size computation. Sample size information is included throughout the figure legends.

**Replicates**

- You should report how often each experiment was performed
- You should include a definition of biological versus technical replication
- The data obtained should be provided and sufficient information should be provided to indicate the number of independent biological and/or technical replicates
- If you encountered any outliers, you should describe how these were handled
- Criteria for exclusion/inclusion of data should be clearly stated
- High-throughput sequence data should be uploaded before submission, with a private link for reviewers provided (these are available from both GEO and ArrayExpress)

Please outline where this information can be found within the submission (e.g., sections or figure legends), or explain why this information doesn’t apply to your submission:

The RNA-sequencing and Nanostring experiments were performed once with biological replicates (n=4 for RNA-sequencing, n=2 or 3 for Nanostring experiments, as indicated). Each data point provided is a biological replicate, defined as separate cell samples within each experiment, with independent experimental replicates performed on separate occasions. For qPCR experiments, each biological replicate is the average of two technical replicates, defined as the same biological sample, measured twice from two distinct reactions. Sequencing reactions of ATF4 knockout lines were performed until a line with two distinct mutations was identified. With the exception of the immunoblots in Figure 2D, which were done once, all remaining experiments were performed at least twice and the number of times an experiment was repeated is reported in the figure legends. RNA-sequencing data was uploaded to GEO under the accession number GSE158605.

**Statistical reporting**

- Statistical analysis methods should be described and justified
- Raw data should be presented in figures whenever informative to do so (typically when N per group is less than 10)
- For each experiment, you should identify the statistical tests used, exact values of N, definitions of center, methods of multiple test correction, and dispersion and precision measures (e.g., mean, median, SD, SEM, confidence intervals; and, for the major substantive results, a measure of effect size (e.g., Pearson's r, Cohen's d)
- Report exact p-values wherever possible alongside the summary statistics and 95% confidence intervals. These should be reported for all key questions and not only when the p-value is less than 0.05.

Please outline where this information can be found within the submission (e.g., sections or figure legends), or explain why this information doesn’t apply to your submission:

Sample sizes are reported in the figure legends. Statistical tests are reported in the figure legends and expanded upon in the statistics section of the methods. P-values are denoted in the figures and reported in the figure legends.

(For large datasets, or papers with a very large number of statistical tests, you may upload a single table file with tests, Ns, etc., with reference to sections in the manuscript.)

**Group allocation**

- Indicate how samples were allocated into experimental groups (in the case of clinical studies, please specify allocation to treatment method); if randomization was used, please also state if restricted randomization was applied
- Indicate if masking was used during group allocation, data collection and/or data analysis

Please outline where this information can be found within the submission (e.g., sections or figure legends), or explain why this information doesn’t apply to your submission:

No grouping, randomization, or masking was used.

**Additional data files (“source data”)**

- We encourage you to upload relevant additional data files, such as numerical data that are represented as a graph in a figure, or as a summary table
- Where provided, these should be in the most useful format, and they can be uploaded as “Source data” files linked to a main figure or table
- Include model definition files including the full list of parameters used
- Include code used for data analysis (e.g., R, MatLab)
- Avoid stating that data files are “available upon request”

Please indicate the figures or tables for which source data files have been provided:

Supplementary table 1 includes the list of RNA-sequencing genes from each category of the Venn diagram shown in Figure 1C. Supplementary table 2 includes the ISR-only gene list used for the CiiiDER analysis and the results of the CiiiDER analysis shown in Figure 2A. Supplementary table 3 includes the heat map values for each Nanostring experiment in Figure 3. RNA sequencing data is available at GEO with accession number GSE158605.
